# Expression of *ustR* and the Golgi protease KexB are required for ustiloxin B biosynthesis in *Aspergillus oryzae*

**DOI:** 10.1186/s13568-016-0181-4

**Published:** 2016-02-03

**Authors:** Akira Yoshimi, Myco Umemura, Nozomi Nagano, Hideaki Koike, Masayuki Machida, Keietsu Abe

**Affiliations:** ABE-project, New Industry Creation Hatchery Center, Tohoku University, 6-6-10 Aoba, Aramaki, Aoba-ku, Sendai, Miyagi 980-8579 Japan; Bioproduction Research Institute, National Institute of Advanced Industrial Science and Technology (AIST), 17-2-1 Higashi-Nijo, Tsukisamu, Toyohira-ku, Sapporo, Hokkaido 062-8517 Japan; Biotechnology Research Institute for Drug Discovery, National Institute of Advanced Industrial Science and Technology (AIST), 2-4-7 Aomi, Koto-ku, Tokyo 135-0064 Japan; Bioproduction Research Institute, National Institute of Advanced Industrial Science and Technology (AIST), 1-1-1 Higashi, Tsukuba, Ibaraki 305-8566 Japan; Laboratory of Applied Microbiology, Department of Microbial Biotechnology, Graduate School of Agricultural Sciences, Tohoku University, 1-1 Amamiya, Tsutsumi-dori, Sendai, Miyagi 981-8555 Japan

**Keywords:** *Aspergillus oryzae*, Ustiloxin B, Secondary metabolite, Fungal C6-type transcription factor, RiPS pathway

## Abstract

Ustiloxin B, originally isolated from the fungus *Ustilaginoidea virens*, is a known inhibitor of microtubule assembly. Ustiloxin B is also produced by *Aspergillus flavus* and is synthesized through the ribosomal peptide synthesis pathway. In *A. flavus*, the gene cluster associated with ustiloxin B production contains 15 genes including those encoding a fungal C6-type transcription factor and ustiloxin B precursor. Although the koji mold *Aspergillus oryzae*, which is genetically close to *A. flavus*, has the corresponding gene cluster, it does not produce ustiloxin B, which may be explained by the fact that the gene encoding the transcription factor UstR is not expressed. Here, to investigate whether ustiloxin B can be produced by expressing *ustR* in *A. oryzae*, we constructed *ustR* expression (*ustR*^EX^) strains and analyzed ustiloxin B production. In the *ustR*^EX^ strains, all genes in the cluster were up-regulated, in line with expression of *ustR*, and ustiloxin B produced. To elucidate whether the KexB protease is involved in the processing of the ustiloxin B precursor protein UstA, which has repeats of basic amino acid doublets resembling KexB target sites, we also constructed a *ustR*^EX^ strain with the ∆*kexB* genotype. Although *ustR* was expressed in this strain, ustiloxin B was barely detectable. This finding strongly suggests that KexB is required for ustiloxin B production.

**Electronic supplementary material:**

The online version of this article (doi:10.1186/s13568-016-0181-4) contains supplementary material, which is available to authorized users.

## Introduction

The koji mold *Aspergillus oryzae* is an important filamentous fungus used in the traditional Japanese fermentation industry to produce *sake* (rice wine), *shoyu* (soy sauce), and *miso* (soybean paste) (Machida et al. 2008). Filamentous fungi are generally able to secrete large amounts of various hydrolytic enzymes. *Aspergillus oryzae* has a high potential for secretion of industrially important enzymes such as amylases and proteases (Kobayashi et al. 2007; Machida et al. 2008). In addition, its long history of extensive use in the food industry has placed *A. oryzae* on the list of Generally Recognized as Safe (GRAS) organisms compiled by the Food and Drug Administration (FDA) in the USA (Abe et al. 2006). The safety of this organism is also supported by the World Health Organization (WHO) (FAO/WHO 1987). Although *A. oryzae* is genetically very close to *Aspergillus flavus*, which produces many secondary metabolites, including the most potent natural carcinogen aflatoxin and the tremorgenic mycotoxin aflatrem, there is no record of *A. oryzae* producing any toxic metabolites because its secondary metabolite genes are silenced (Machida et al. 2005). For instance, the *A. oryzae* homologs of the aflatoxin biosynthesis gene cluster are not expressed even under conditions that are favorable for aflatoxin production in *A. flavus* and *Aspergillus parasiticus* (Kusumoto et al. 1998; Watson et al. 1999; Takahashi et al. 2002; Zhang et al. 2005). Therefore, *A. oryzae* may be a suitable host for production of not only heterologous proteins but also secondary metabolites with important medical activities (Kobayashi et al. 2007; Machida et al. 2008; Sakai et al. 2008).

Ustiloxin B is a toxic cyclic peptide that was originally identified in *Ustilaginoidea virens*, a pathogenic fungus affecting rice (Koiso et al. 1992, 1994, 1998). Recently, the ustiloxin B biosynthetic gene cluster was identified in *A. flavus* using a novel method to predict gene clusters from transcriptome data, and subsequently validated by LC-MS analysis of ustiloxin B production by gene deletion mutants (Umemura et al. 2013, 2014). Although ustiloxin B production by *A. oryzae* is undetectable under normal growth conditions, the corresponding gene cluster is present in the *A. oryzae* genome (Machida et al. 2005; Umemura et al. 2013); it contains 15 genes, including *ustR*, which encodes a fungal-type Zn(II)_2_Cys_6_ (C6) transcription factor (Umemura et al. 2013). Interestingly, the upstream region of *ustR*, which might be involved in the transcriptional regulation of *ustR*, is deleted in *A. oryzae* (Umemura et al. 2013).

Fungal secondary metabolites are biosynthesized by proteins encoded by clusters of coordinately regulated genes, and most of these clusters encode enzymes, such as polyketide synthase (PKS) and non-ribosomal peptide synthase (NRPS), which catalyze condensation reactions of monomeric units to form oligomeric intermediates. Ustiloxin B synthesis does not involve PKS or NRPS: this is the first case of a ribosomally synthesized peptide in a filamentous fungus (Umemura et al. 2014). Ustiloxin B consists of tetrapeptides, Tyr-Ala-Ile-Gly (YAIG). It is circularized at the side chains of Tyr and Ile, and modified with a methyl group, a hydroxyl group, and the non-protein-coding amino acid norvaline; all three modifications are at the tyrosine (Umemura et al. 2014). Since the protein encoded by *ustA* contains 16 repeats of YAIG, UstA is thought to be the precursor of ustiloxin B (Umemura et al. 2014). The *U. virens* gene for the precursor (UstA) has five Tyr-Val-Ile-Gly (YVIG) and three YAIG motifs, corresponding to the sequences of ustiloxin A and ustiloxin B, respectively (Koiso et al. 1992, 1994; Tsukui et al. 2015). Thus, ustiloxins produced by *U. virens* (both are cyclic peptides) are probably synthesized via the ribosomal peptide synthesis (RiPS) pathway, as in the case of *A. flavus* ustiloxin B (Tsukui et al. 2015).

In both *A. flavus* and *U. virens*, the UstA protein possesses an N-terminal signal peptide for import into the endoplasmic reticulum, followed by a novel repeating sequence containing basic amino acid doublets, KR, which resemble the target sites of the subtilisin-like endoprotease Kex2 from *Saccharomycescerevisiae* (Mizuno et al. 1988; Fuller et al. 1989). Kex2 of *S. cerevisiae* is a Ca^2+^-dependent transmembrane serine protease that cleaves secretory proproteins at the carboxyl side of KR and RR in a late Golgi compartment (Fuller et al. 1989; Redding et al. 1991). Therefore, the UstA proteins may be processed in the Golgi apparatus at the C-terminal side of KR by the KexB protease, which is an *A. oryzae* ortholog of *S. cerevisiae* Kex2 (Mizutani et al. 2004). However, the relationship between the biosynthesis of RiPS compounds, such as ustiloxin B, and the KexB protease has not been characterized directly.

In the present study, we constructed several *ustR* expression (*ustR*^EX^) mutants of *A. oryzae* to determine whether they would be able to produce ustiloxin B, in contrast to the wild-type strain. Analyses of the transcription of ustiloxin B biosynthetic cluster genes in these strains revealed that *ustR* expression induced the expression of all other genes in the cluster and ustiloxin B production in the *ustR*^EX^ strains. Because *ustR* expression and ustiloxin B production were never detected in the wild-type strain, *A. oryzae* might silence *ustR*, resulting in the lack of ustiloxin B synthesis. The involvement of the KexB protease in the processing of the precursor protein, UstA, was validated by the analysis of ustiloxin B production in a *ustR*^EX^ strain with *kexB* deletion (*ustR*^EX^/∆*kexB*). We propose that the expression of the transcription factor *ustR* is critical for the production of ustiloxin B in *A. oryzae*, and that the KexB endopeptidase is involved in UstA processing.

## Methods

### Strains and growth media

*Aspergillus oryzae* strains used in this study are listed in Table 1. RIB40 was used as the wild-type strain; ∆*ligD::sC*, ∆*kexB* and NSlD-∆P10 were used as parents to construct *ustR*-expressing (*ustR*^EX^) strains. These strains were grown in Czapek-Dox (CD) minimal medium or CDE medium, which is CD medium supplemented with 70 mM monosodium glutamate instead of sodium nitrate (NaNO_3_) as a nitrogen source for preparation of conidial suspension (Mizutani et al. 2008). CDE medium containing 2 % maltose as a carbon source (designated CDEm medium) or YPM medium (1 % yeast extract, 2 % polypeptone, 2 % maltose) was used for up-regulation of *glaA142* promoter-driven *ustR* in the *ustR*^EX^ strains. To analyze gene transcription and to measure ustiloxin B production, strains were cultured in V8-juice liquid medium referred to hereafter as V8 medium [20 % (v/v) V8 juice (Campbell’s, Camden, NJ, USA) with 0.3 % CaCO_3_] at 30 °C on a rotary shaker at 160 rpm.

**Table 1:** Strains used in this study

Strain	Parental strain	Genotype	Source or reference
RIB40		Wild type	Machida et al. (2005)
∆*ligD::sC*	NS4^a^	*sC* ^-^, *niaD* ^-^, ∆*ligD::sC*	Mizutani et al. (2008)
*ustR* ^EX^-G101	∆*ligD::sC*	*sC*, *niaD* ^-^, ∆*ligD::sC*, P*glaA142*-*ustR::niaD*	This study
*ustR* ^EX^-G301	∆*ligD::sC*	*sC*, *niaD* ^-^, ∆*ligD::sC*, P*glaA142*-*ustR::niaD*	This study
∆*kexB*	niaD300^b^	*niaD* ^-^, ∆*kexB::ptrA*	Kindly provided by Dr. Y. Yamagata
*ustR* ^EX^/∆*kexB*	∆*kexB*	*niaD* ^-^, ∆*kexB::ptrA*, P*glaA142*-*ustR::niaD*	This study
NSlD-∆P10	NSPlD-tApEnBdIVdVaApApAapAd1	*sC*, *niaD* ^-^, *adeA* ^-^, ∆*argB::adeA* ^-^, ∆*ligD::argB*, ∆*pyrG::adeA*, ∆*tppA*, ∆*pepE*, ∆*nptB*, ∆*dppIV*, ∆*dppV*, ∆*alpA*, ∆*pepA*, ∆*AopepAa*, ∆*AopepAd*, ∆*cpI::pyrG*	Yoon et al. (2011)
*ustR* ^EX^/∆P10	NSlD-∆P10	*sC*, *niaD* ^-^, *adeA* ^-^, ∆*argB::adeA* ^-^, ∆*ligD::argB*, ∆*pyrG::adeA*, ∆*tppA*, ∆*pepE*, ∆*nptB*, ∆*dppIV*, ∆*dppV*, ∆*alpA*, ∆*pepA*, ∆*AopepAa*, ∆*AopepAd*, ∆*cpI::pyrG*, P*glaA142*-*ustR::niaD*	This study

^a^Yamada et al. (1997)

^b^Minetoki et al. (1996)

### Construction of *ustR* expression mutants in *A. oryzae*

The plasmid for *ustR* expression was constructed as follows. The *A. oryzae ustR* gene was amplified using KOD-plus DNA polymerase (Toyobo, Osaka, Japan) and the primers ustR-MCS-F and ustR-MCS-R (Additional file 1: Table S1). Each primer was designed to introduce a *Not*I site. *Aspergillusoryzae* RIB40 genomic DNA was used as the template. The amplified fragment was digested with *Not*I and inserted into the *Not*I site of pNGA142 (Minetoki et al. 1998, 2003), which contained the *glaA142* promoter, *agdA* terminator, and *niaD* gene as a selectable marker in *A. oryzae*. The resulting plasmid (pNGAustR) was used to transform the ∆*ligD::sC*, ∆*kexB*, and NSlD-∆P10 strains and was integrated into their genomes as described previously (Fujioka et al. 2007). Two mutant lines of *ustR*^EX^, designated G101 and G301, were obtained by transformation of the parental strain ∆*ligD::sC* strain; both G101 and G301 were used in the following experiments.

### Analysis of the transcription levels of the ustiloxin B gene cluster by quantitative RT-PCR

Real-time RT-PCR was performed as described previously (Yoshimi et al. 2013, 2015). Primer sets for quantifying the expression of the ustiloxin B cluster genes are listed in Additional file 1: Table S1. The histone H2B gene was used as a normalization reference (internal control) for target gene expression ratios. A sample of unmanipulated cells of the control strain (∆*ligD::sC*) cultured in V8 medium was set as a calibrator in each experiment. Statistical analyses were performed using Welch’s *t* test (*p* < 0.01 was considered significant).

### Production of ustiloxin B

Three replicates of the control and each *ustR*^EX^ strain were cultivated in 100 mL of V8 medium in 200-mL Erlenmeyer flasks at 30 °C and 160 rpm. After 1, 3, 5, and 7 days, 10-mL culture aliquots were harvested and the equivalent volume of acetone was added. The mixtures were incubated for 1 h at room temperature, and the mycelia were removed by filtration through Miracloth. Each filtrate was stirred with an equal amount of ethyl acetate for 1 h at room temperature, centrifuged at 13,000×*g* for 10 min, and the water layer was collected. A 3-μL aliquot of each water extract was separated using an UPLC-MS system (Acquity UPLC I-Class, Xevo G2 QTof, Waters Corp., MA, Milford, USA) equipped with a reversed-phase column (2.1 × 150 mm; Acquity UPLC BEH C18; Waters Corp.) Peptides were eluted with a water–acetonitrile gradient containing 0.1 % formic acid (98:2 for 0.5 min, to 70:30 for 10 min) at a flow rate of 0.4 mL/min. The production of ustiloxin B (C_26_H_39_N_5_O_12_S) was quantified by calculating the peak area of extracted ion chromatograms (EICs) of m/z 646.239 ± 0.03 [M + H]^+^ at the retention time of 3.1 min. An authentic sample of ustiloxin B (Umemura et al. 2013) was used for external calibration with the QuanLynx software (version 4.1, Waters Corp.).

## Results

### Expression of *ustR* in *A. oryzae*

**Fig. 1:**
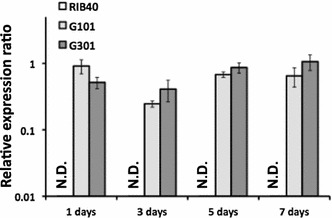
Expression of the *ustR* gene in wild-type (RIB40) and *ustR* ^EX^ (G101 and G301) *A. oryzae* strains. The strains were grown in V8 medium at 30 °C. Quantitative RT-PCR was used to determine the levels of transcription of the *ustR* gene and was performed on total RNA with *ustR*-specific primers (Additional file 1: Table S1). Each value represents the ratio of *ustR* expression to that of the histone H2B gene in each strain. *Error bars* represent standard deviations (*n* = 3). *ND* not detectable

In the wild-type strain, no transcript of the *ustR* gene was detected at the time points examined (Fig. 1), whereas in the *ustR*^EX^ strains the *ustR* transcript was detectable in CDEm (data not shown) and V8 media (Fig. 1). There were no notable differences between the two independent *ustR*^EX^ mutant lines (G101 and G301; Fig. 1).

**Fig. 2:**
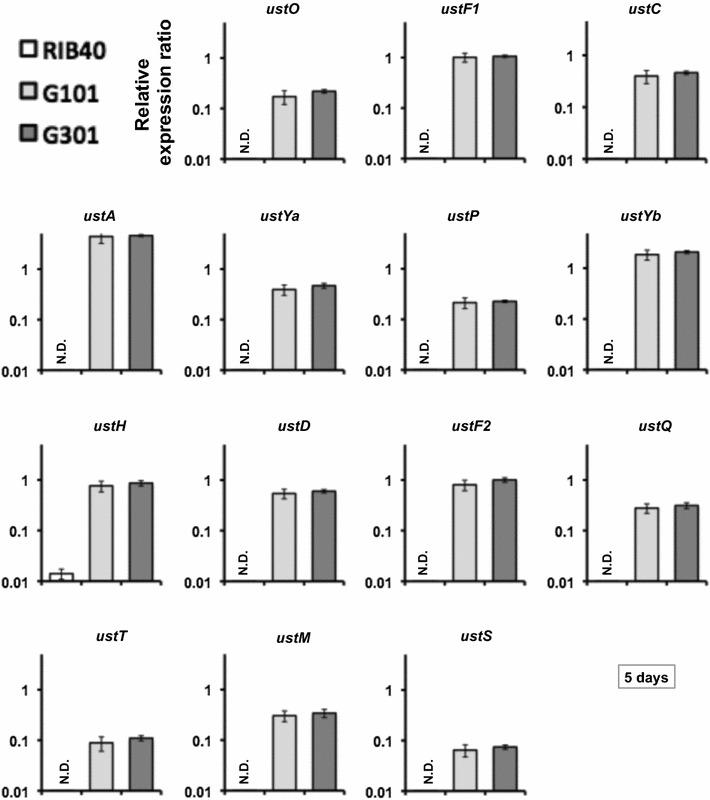
Expression of the genes of the cluster for ustiloxin B biosynthesis in *A. oryzae* wild-type (RIB40) and *ustR* ^EX^ (G101 and G301) strains. The strains were grown in V8 medium at 30 °C for 5 days. Quantitative RT-PCR was used to determine the levels of transcription and was performed on total RNA with gene-specific primers (Additional file 1: Table S1). Each value represents the ratio of expression to that of the histone H2B gene in each strain. *Error bars* represent standard deviations (*n* = 3). *ND* not detectable

Next, we analyzed the expression of the other 14 genes in the ustiloxin B biosynthetic cluster to test whether it is affected by *ustR* expression. Whereas the transcripts of all these genes (except *ustH*) were undetectable in the wild-type strain, they were strongly induced in both *ustR*^EX^ strains grown in V8 medium for 5 days (Fig. 2). The transcripts of all genes were present from day 1 until at least 7 days after inoculation (Fig. 2; Additional file 1: Figures S1, S2, S3), in line with the expression of *ustR* (Fig. 1). The co-expression of *ustR* and other genes of this cluster was also detected in CDEm and YPM liquid media (data not shown). These results suggest that the C6-type transcription factor UstR regulates all genes in the ustiloxin B biosynthetic cluster.

### Production of ustiloxin B in the *ustR*^EX^ mutants

**Fig. 3:**
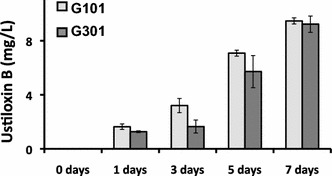
Production of ustiloxin B by *A. oryzae* *ustR* ^EX^ (G101 and G301) strains. The strains were grown in V8 medium at 30 °C. Culture aliquots (10 mL) were harvested at the indicated time points and the amount of ustiloxin B in the culture medium was determined by LC-MS analysis. *Error bars* represent standard deviations (*n* = 3)

To investigate whether *ustR* expression induces ustiloxin B production, we assessed the presence of ustiloxin B in both *ustR*^EX^ strains, G101 and G301. Although the genes in the ustiloxin B cluster were transcribed in CDEm or YPM media in the *ustR*^EX^ strains, the production of ustiloxin B was not detected (data not shown). However, ustiloxin B was detected in V8 medium culture, and its concentration increased continuously at least from 1 to 7 days (Fig. 3). These results indicate that ustiloxin B can be produced in *A. oryzae*, and that *ustR* expression and consequent induction of the genes of the ustiloxin B biosynthetic cluster are responsible for its production.

### Involvement of the KexB protease in ustiloxin B biosynthesis in *A. oryzae*

**Fig. 4:**
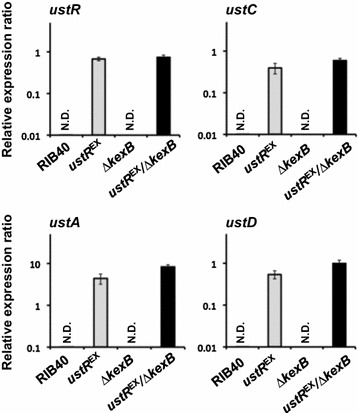
Expression of the genes of the cluster for ustiloxin B biosynthesis in *A. oryzae* wild-type (RIB40), *ustR* ^EX^, ∆*kexB*, and *ustR* ^EX^/∆*kexB* strains. The strains were grown in V8 medium at 30 °C for 5 days. Quantitative RT-PCR was used to determine the levels of transcription of the *ustR* gene and the indicated representative genes; RT-PCR was performed on total RNA with gene-specific primers (Additional file 1: Table S1). Each value represents the ratio of expression relative to that of the histone H2B gene in each strain. *Error bars* represent standard deviations (*n* = 3). *ND* not detectable

**Fig. 5:**
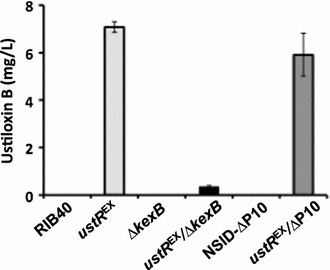
Production of ustiloxin B by *A. oryzae* wild-type (RIB40), *ustR* ^EX^, ∆*kexB*, *ustR* ^EX^/∆*kexB*, NSlD-∆P10, and *ustR* ^EX^/∆P10 strains. The strains were grown in V8 medium at 30 °C for 5 days. Culture aliquots (10 mL) were harvested and the amount of ustiloxin B in the culture medium was determined by LC-MS analysis. *Error bars* represent standard deviations (*n* = 3)

To investigate whether the precursor protein UstA was processed by KexB, we used the *ustR*^EX^ mutant lacking this protease (*ustR*^EX^/*∆kexB*). The transcription levels of the ustiloxin B biosynthetic genes and *ustR* were similar in the *ustR*^EX^/*∆kexB* and *ustR*^EX^ strains grown in V8 medium (Figs. 2, 4; Additional file 1: Figure S4). We then analyzed ustiloxin B production in both *ustR*^EX^ strains (*ustR*^EX^ and *ustR*^EX^/*∆kexB*) and the corresponding control strains (wild-type and *∆kexB*) (Table 1). After 5 days of culture in V8 medium, ustiloxin B was not detectable in the control strains; a considerable amount of ustiloxin B (approximately 7 mg/L) was produced by the *ustR*^EX^ strain, but ustiloxin B was barely detectable in the *ustR*^EX^/∆*kexB* strain (approximately 0.35 mg/L; Fig. 5).

We also generated a *ustR*^EX^ mutant in the NSlD-∆P10 strain, in which the genes encoding 10 secreted proteases of *A. oryzae* are disrupted (Yoon et al. 2011). The *ustR*^EX^/∆P10 strain produced the same level of ustiloxin B as the *ustR*^EX^ strain, whereas the parental strain, NSlD-∆P10, did not produce ustiloxin B (Fig. 5). These results suggest that KexB is critical for ustiloxin B production and is specifically required for proteolytic processing of the precursor protein UstA.

## Discussion

In the present study, we analyzed the transcription of the ustiloxin B gene cluster and the production of ustiloxin B in three types of *ustR*^EX^ mutants of *A. oryzae* and their parental strains (wild-type *A. oryzae* RIB40, ∆*kexB*, and ∆P10) (Table 1). In the wild-type strain, we detected neither the transcripts of any genes of the cluster nor ustiloxin B. In contrast, expression of the *ustR* gene (which encodes a fungal-type C6 transcription factor) from this cluster (Umemura et al. 2014) up-regulated the transcription of all other genes of the cluster and induced ustiloxin B synthesis (Figs. 1, 2, 3). Fungal-type Zn(II)_2_Cys_6_ (C6) transcription factors regulate genes involved in the production of secondary metabolites in filamentous fungi (Payne et al. 1993; Chang et al. 1995; Brown et al. 1996; Marui et al. 2011). One of the best-studied examples is AflR of *A. flavus*, *A. parasiticus*, and *A. nidulans*, which regulates the expression of genes required for the production of aflatoxin and sterigmatocystin (Payne et al. 1993; Chang et al. 1995; Brown et al. 1996). In *A. oryzae*, fungal-type C6 transcription factors regulate genes necessary for the degradation of complex polysaccharides such as starch and xylan. For example, AmyR regulates the expression of the clustered amylolytic genes *agdA*, which encodes α-glucosidase, and *amyA*, which encodes α-amylase (Gomi et al. 2000). In *A. oryzae*, XlnR regulates the expression of more than 30 xylanolytic and cellulolytic genes involved in the degradation of β-1,4-xylan, arabinoxylan, cellulose, and xyloglucan (Noguchi et al. 2009), and ManR regulates the expression of the endo-β-mannanase gene (Ogawa et al. 2012). In contrast, little is known about the role of fungal-type C6 transcription factors in the regulation of the expression of the genes involved in production of secondary metabolites synthesized through the RiPS pathway in *A. oryzae*.

The *A. oryzae* genome contains many gene clusters likely involved in biosynthesis of secondary metabolites, and these clusters are highly enriched in non-syntenic blocks (NSBs) (Machida et al. 2005, 2008; Kobayashi et al. 2007). The analyses of ESTs and DNA microarrays of *A. oryzae* grown under several conditions showed that the transcription levels of the NSB genes are considerably lower than those of the genes in syntenic blocks (Machida et al. 2005; Akao et al. 2007; Tamano et al. 2008). The absence of toxin production in *A. oryzae* is thought to be attributable to silencing of the secondary metabolite biosynthetic genes (Barbesgaard et al. 1992; Machida et al. 2008; Tokuoka et al. 2008). *Aspergillus oryzae* may possess a silencing mechanism similar to that observed in the regulation of aflatoxin biosynthesis in *Aspergillus sojae*, which is closely related to *A. oryzae* and is also known as koji mold. Silencing is thought to be caused by the lack of *aflR* expression and/or by non-functional AflR in *A. sojae* (Matsushima et al. 2001a, b). AflR regulates transcription of a gene cluster for aflatoxin biosynthesis in *Aspergillus* species (Woloshuk et al. 1994). Although the *aflR* gene is present within the aflatoxin biosynthesis gene cluster, *aflR* is not expressed or AflR is non-functional in *A. oryzae* and *A. sojae* (Kusumoto et al. 1998; Watson et al. 1999; Takahashi et al. 2002). The mechanism of *ustR* expression silencing may be similar to that of *aflR* expression silencing in *A. oryzae*.

The deletion of the upstream region of *ustR* might silence the ustiloxin B gene cluster in *A. oryzae*, resulting in the lack of ustiloxin B production even under conditions that are favorable for ustiloxin B production in *A. flavus*. However, some wild-type *A. oryzae* strains have no deletion in the *ustR* upstream region and are in this respect similar to *A. flavus* (our unpublished data). Therefore, this deletion is strain-specific in *A. oryzae* and might not affect ustiloxin B production. *Aspergillus oryzae* has orthologs of VelB, VeA, and LaeA (Marui et al. 2010), which are global regulators of secondary metabolic genes in *Aspergillus* species (Bok and Keller 2004; Bayram et al. 2008; Amare and Keller 2014). The absence of *aflR* expression was found in the ∆*laeAA. oryzae* strain (Ken Oda, personal communication), suggesting that *aflR* expression is regulated by LaeA in *A. oryzae*, as in other *Aspergillus* species. Thus, another possible explanation of ustiloxin B gene cluster silencing in *A. oryzae* is that global regulators of secondary metabolic genes such as LaeA might regulate *ustR* expression.

Although the expression of the *ustR* gene and the induction of the cluster genes were confirmed in the *ustR*^EX^ strain, the production of ustiloxin B was not detected in CDEm liquid medium, in which the *ustR*^EX^ strain was grown. Similar results were obtained in *A. flavus* (our unpublished data). Ustiloxin B production was also not detected in the nutritionally rich YPM liquid medium (our unpublished data). Ustiloxin B was produced by the *ustR*^EX^ strain only in V8 medium or cracked-maize medium (Fig. 3 and data not shown). These data suggest that the amounts of amino acids were insufficient for ustiloxin B production in CDEm medium or that some essential factor(s), such as vitamin(s), derived from vegetables or maize is(are) needed for ustiloxin B production by *A. oryzae* even in the presence of sufficient amounts of amino acids in YPM medium.

Our data revealed that KexB endopeptidase is required for the processing of UstA, the ustiloxin B precursor, which contains a sequence with signal peptide characteristics and was predicted to be processed by the subtilisin-like endoprotease Kex2 (Umemura et al. 2014). However, a low level of ustiloxin B production was still detectable in the *ustR*^EX^/*∆kexB* strain. This residual ustiloxin B production can be explained as follows: (1) in the absence of KexB, the expression of *ustA* was still up-regulated by the expression of *ustR*, resulting in sufficient amounts of UstA; (2) a small amount of UstA could be processed non-specifically by some protease(s) in the Golgi apparatus and produce low levels of ustiloxin B; (3) subsequent biosynthetic steps of ustiloxin B production could progress regardless of the presence or absence of KexB. A recent study on the biosynthesis of RiPS compounds in other fungal species predicted that the precursor protein of novel cyclic oligopeptides is likely to be processed by the subtilisin-like endoprotease in the ascomycetes *Epichloë* (Johnson et al. 2015). Our study provides the first direct evidence that a subtilisin-like endoprotease is involved in the biosynthesis of a RiPS compound (ustiloxin B) in filamentous fungi.

Since the *A. oryzae* genome encodes a number of various proteases (Machida et al. 2005; Kobayashi et al. 2007), the identification of the protease potentially involved in UstA processing in the absence of KexB is quite difficult. However, ustiloxin B production was not altered in the *ustR*^EX^/∆P10 strain (Fig. 5), which expresses *ustR* and in which genes encoding 10 secreted proteases (TppA, PepE, NptB, DppIV, DppV, AlpA, PepA, AoPepAa, AoPepAd, and CpI) are disrupted (Table 1) (Yoon et al. 2011). Therefore, none of these 10 proteases is involved in UstA processing. Further studies are necessary for elucidation of the biosynthetic pathway of ustiloxin B in *A. oryzae*.

## Additional file

10.1186/s13568-016-0181-4 PCR primers used in this study.** Figure S1.** Expression of the genes of the cluster for ustiloxin B biosynthesis in *A. oryzae* wild-type (RIB40) and *ustR* ^EX^ (G101 and G301) strains grown for 1 day. **Figure S2. **Expression of the genes of the cluster for ustiloxin B biosynthesis in *A. oryzae* wild-type (RIB40) and *ustR* ^EX^ (G101 and G301) strains grown for 3 day. **Figure S3.** Expression of the genes of the cluster for ustiloxin B biosynthesis in *A. oryzae* wild-type (RIB40) and *ustR* ^EX^ (G101 and G301) strains grown for 7 day. **Figure S4.** Expression of the genes of the cluster for ustiloxin B biosynthesis in the *A. oryzae* strains ∆*kexB*, *ustR* ^EX^/∆*kexB*, NSlD-∆P10 (∆*P10*), and *ustR* ^EX^/∆P10.
