# Supplementary material for: Expression of ustR and the Golgi protease KexB are required for ustiloxin B biosynthesis in Aspergillus oryzae
Source: AMB Express. 2016 Feb 3;6:9. doi: 10.1186/s13568-016-0181-4 (PMC4740483; doi:10.1186/s13568-016-0181-4)
Supplement: Supplementary file 1 — 10.1186/s13568-016-0181-4 PCR primers used in this study. Figure S1. Expression of the genes of the cluster for ustiloxin B biosynthesis in A. oryzae wild-type (RIB40) and ustR EX (G101 and G301) strains grown for 1 day. Figure S2. Expression of the genes of the cluster for ustiloxin B biosynthesis in A. oryzae wild-type (RIB40) and ustR EX (G101 and G301) strains grown for 3 day. Figure S3. Expression of the genes of the cluster for ustiloxin B biosynthesis in A. oryzae wild-type (RIB40) and ustR EX (G101 and G301) strains grown for 7 day. Figure S4. Expression of the genes of the cluster for ustiloxin B biosynthesis in the A. oryzae strains ∆kexB, ustR EX/∆kexB, NSlD-∆P10 (∆P10), and ustR EX/∆P10. [file 13568_2016_181_MOESM1_ESM.pdf]

Additional file 1

AMB Express

Expression of *ustR* and the Golgi protease KexB are required for ustiloxin B biosynthesis in *Aspergillus oryzae*

Akira Yoshimi[1], Myco Umemura[2], Nozomi Nagano[3], Hideaki Koike[4], Masayuki Machida[2], Keietsu Abe[1,5]*

[1]ABE-project, New Industry Creation Hatchery Center, Tohoku University, 6-6-10 Aoba, Aramaki, Aoba-ku, Sendai, Miyagi 980-8579, Japan

[2]Bioproduction Research Institute, National Institute of Advanced Industrial Science and Technology (AIST), 17-2-1 Higashi-Nijo, Tsukisamu, Toyohira-ku,Sapporo, Hokkaido 062-8517, Japan

[3]Biotechnology Research Institute for Drug Discovery, National Institute of Advanced Industrial Science and Technology (AIST), 2-4-7 Aomi, Koto-ku, Tokyo 135-0064, Japan

[4]Bioproduction Research Institute, National Institute of Advanced Industrial Science and Technology (AIST), 1-1-1 Higashi, Tsukuba, Ibaraki 305-8566, Japan

[5]Laboratory of Applied Microbiology, Department of Microbial Biotechnology, Graduate School of Agricultural Sciences, Tohoku University, 1-1 Amamiya, Tsutsumi-dori, Sendai 981-8555, Japan

*To whom correspondence should be addressed. Tel and fax: +81-22-795-3205; E-mail: kabe@niche.tohoku.ac.jp

Table S1. PCR primers used in this study

Purpose	Name	Sequence (5′-3′)
ustR cloning		
	ustR-MCS-F	ACAAGCTTGCGGCCGCCACGTGACTAGTATGTCAGGGGCCCAGCGGCGT
	ustR-MCS-R	ACTAGTCACGTGGCGGCCGCTCATATGAACAGGATACTTAAATTTAGC
Quantitative RT PCR		
	Histone-RT-F	CAAGCGTATCTCTGCCATGA
	Histone-RT-R	CACCGAAACCGTAGAGGGTA
	ustR-RT-F	ATCTCCACACCGTTGTCTCT
	ustR-RT-R	GAACAGCTCTCGCTGAGAAC
	ustO-RT-F	ATGGAACAGTAGGAGCCTTG
	ustO-RT-R	TATCCTACCCACTGTGCGAC
	ustF1-RT-F	AATACGTCCGCGACCTTATG
	ustF1-RT-R	TGCTCCTTTCTTCCTTTCATC
	ustC-RT-F	GTGAGGACGCAAACTCGTTC
	ustC-RT-R	GCAGCAACTTTGTACAACTG
	ustA-RT-F	TATCGGCATTGATAAGAAGC
	ustA-RT-R	ATCCTCAACTGATCCTCCAC
	ustYa-RT-F	TGAGACAGGCTATCATGTGT
	ustYa-RT-R	CCATCGAGCAATAGCATCGA
	ustP-RT-F	ACTGACAGGATTCCCAATTC
	ustP-RT-R	TCGGGAAAGACATAGTTCTC
	ustYb-RT-F	GCAGTCAACCAGACGGAGAG
	ustYb-RT-R	GGTGCTCATTGGAATGTGTG
	ustH-RT-F	TCTCCGGTATTGACCAGAAC
	ustH-RT-R	CATGTCACTTCATGCCCTAG
	ustD-RT-F	CAAAGGGTTGCGATTGTGAC
	ustD-RT-R	CCAAACCATCGCTACTCTTC
	ustF2-RT-F	CAGCTACCAACTGAGACTG
	ustF2-RT-R	TTAATCGCGACAAGCTCCTG
	ustQ-RT-F	ATGTCCACCATGCACAACTG
	ustQ-RT-R	CGAGATACCGGGATATCCTC
	ustT-RT-F	TTTTGTGATGTTCCTCGCTG
	ustT-RT-R	GTGAAGACAGTTCCAATGTG
	ustM-RT-F	TCAGGGATGGGAAGGTCAC
	ustM-RT-R	GAAGCGAGGATCTCACTCTC
	ustS-RT-F	GTCGTGGAGTCATCGGTTTG
	ustS-RT-R	AACCACTGCACAGAAGCATG

Not I sites are underlined

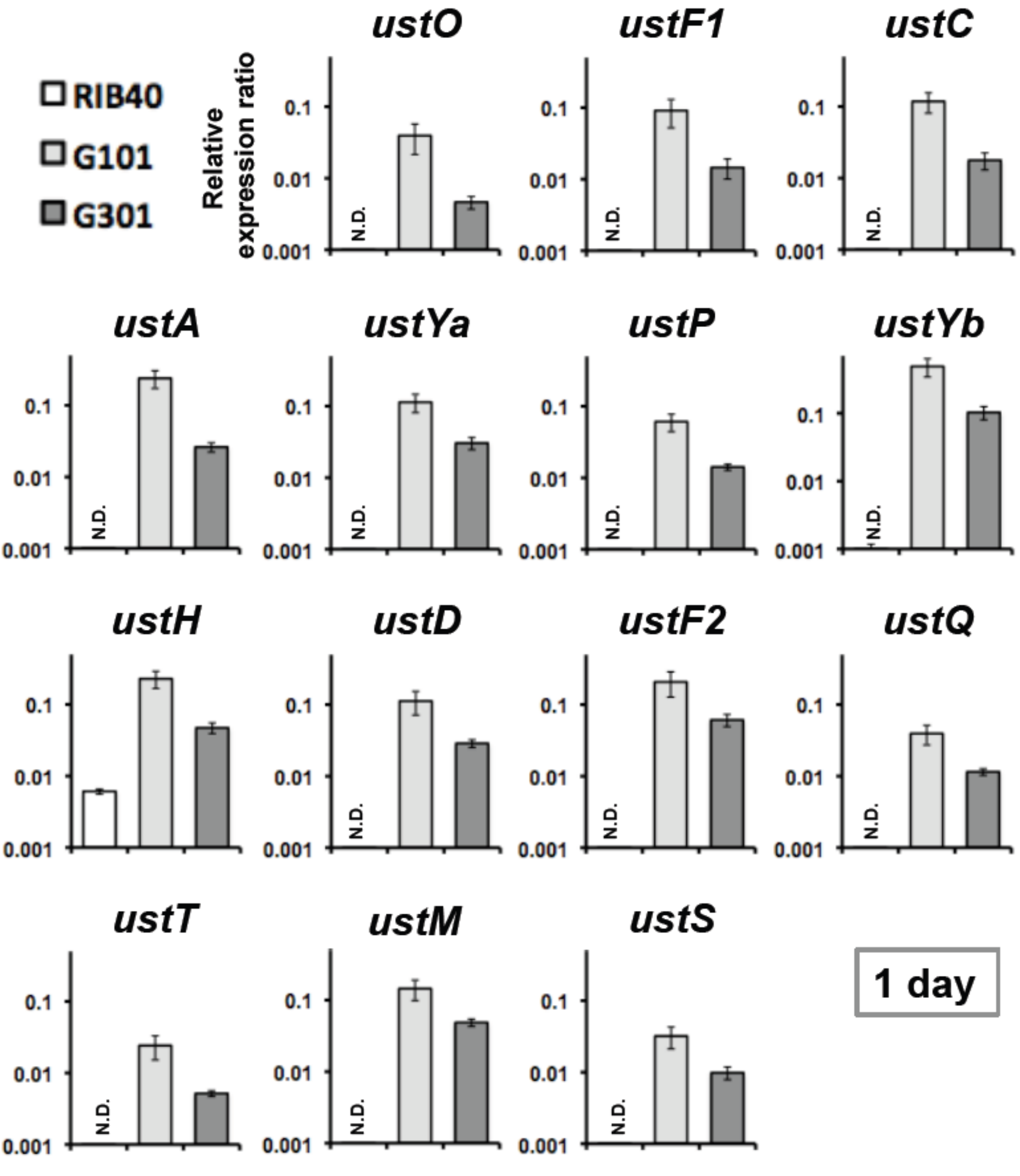

Figure S1 Expression of the genes of the cluster for ustiloxin B biosynthesis in *A. oryzae* wild-type (RIB40) and *ustR*^{EX} (G101 and G301) strains grown for 1 day. The strains were grown in V8 medium at 30 °C. Quantitative RT-PCR was used to determine the levels of transcription of the indicated genes and was performed on total RNA with gene-specific primers (Table S1). Each value represents the ratio of expression to that of the histone H2B gene in each strain. Error bars represent standard deviations (*n*=3). N.D., not detectable.

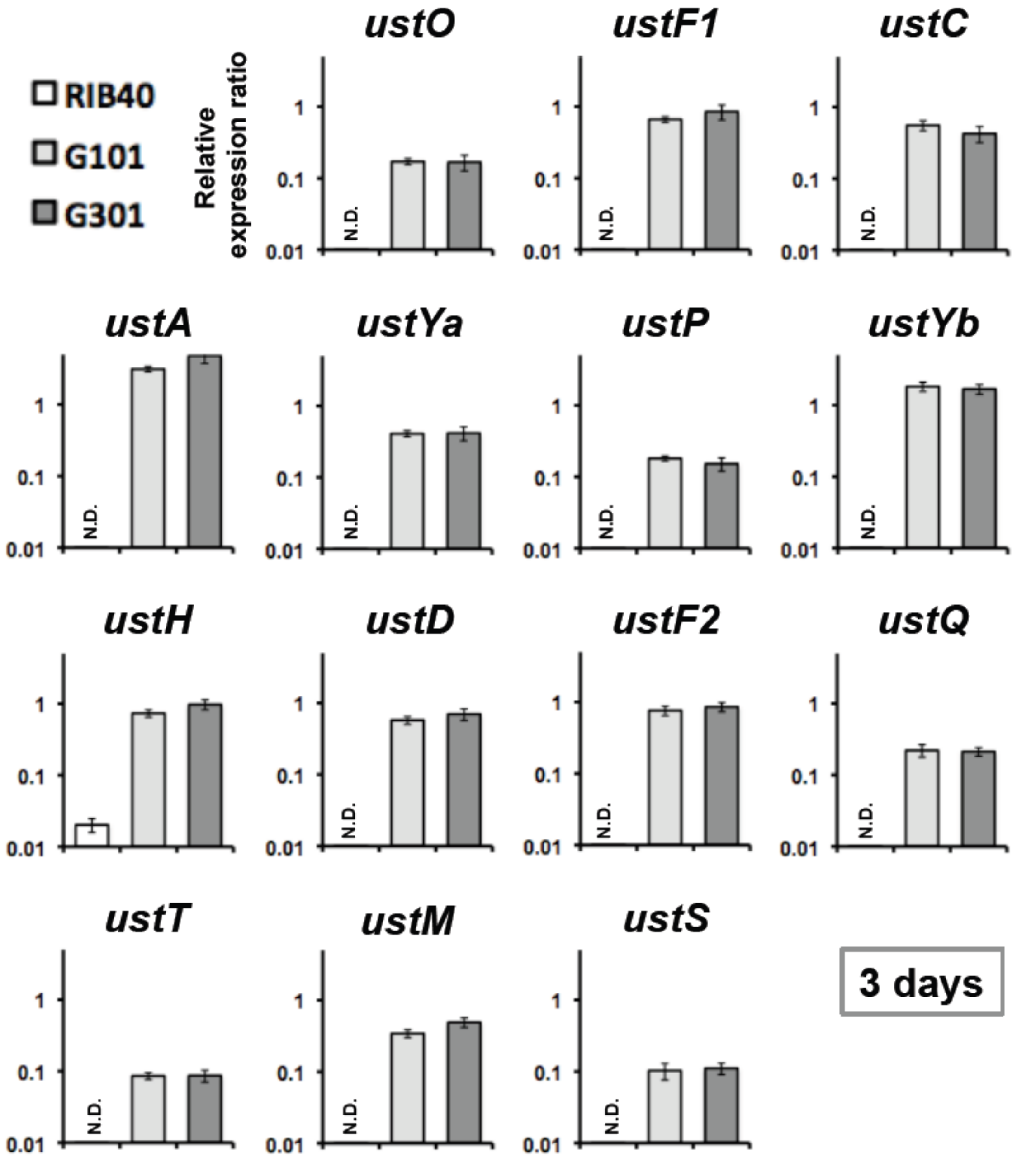

Figure S2 Expression of the genes of the cluster for ustiloxin B biosynthesis in *A. oryzae* wild-type (RIB40) and *ustR*^{EX} (G101 and G301) strains grown for 3 days. The strains were grown in V8 medium at 30 °C. Quantitative RT-PCR was used to determine the levels of transcription of the indicated genes and was performed on total RNA with gene-specific primers (Table S1). Each value represents the ratio of expression to that of the histone H2B gene in each strain. Error bars represent standard deviations (n=3). N.D., not detectable.

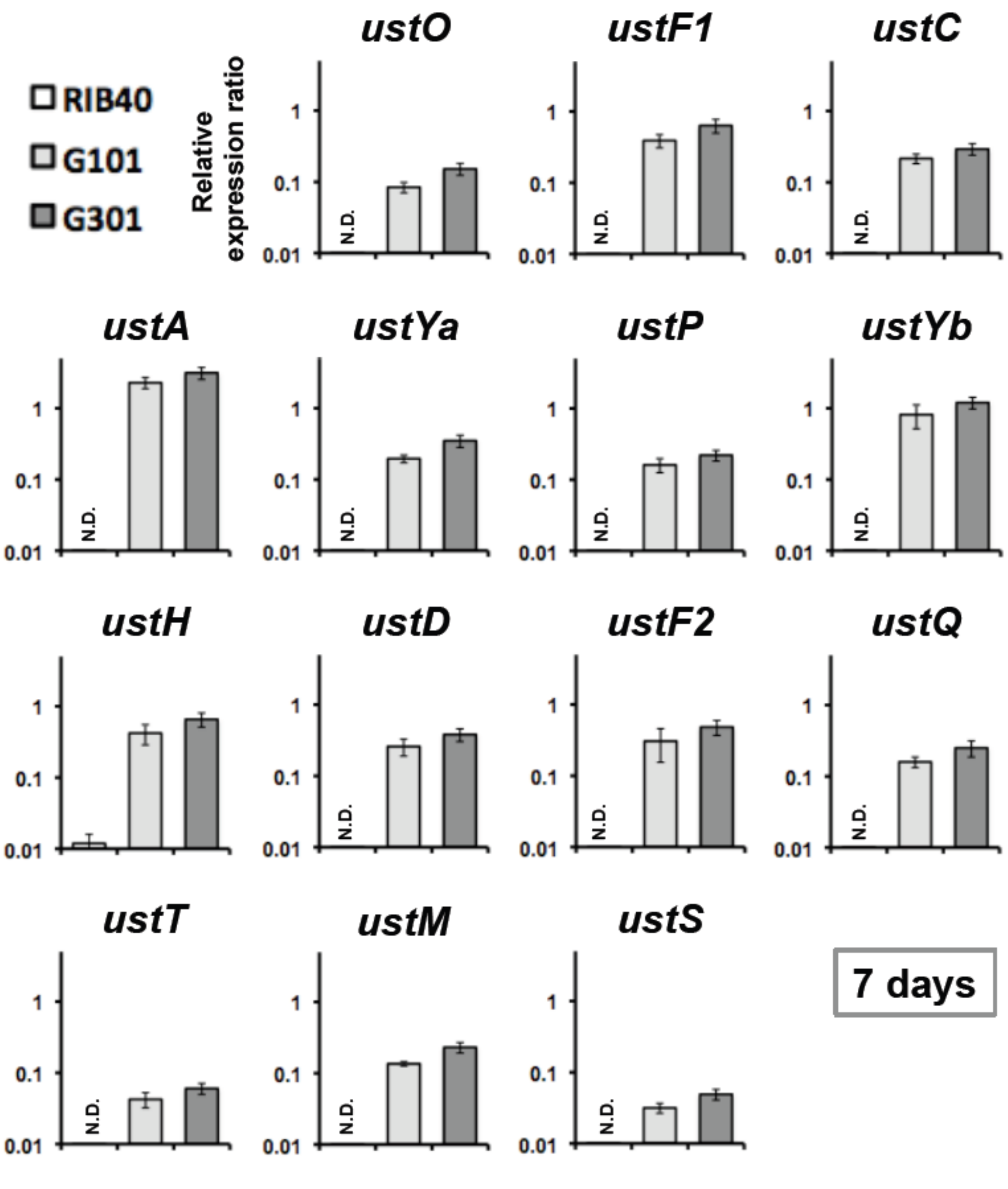

Figure S3 Expression of the genes of the cluster for ustiloxin B biosynthesis in *A. oryzae* wild-type (RIB40) and *ustR*^{EX} (G101 and G301) strains grown for 7 days. The strains were grown in V8 medium at 30 °C. Quantitative RT-PCR was used to determine the levels of transcription of the indicated genes and was performed on total RNA with gene-specific primers (Table S1). Each value represents the ratio of expression to that of the histone H2B gene in each strain. Error bars represent standard deviations (*n*=3). N.D., not detectable.

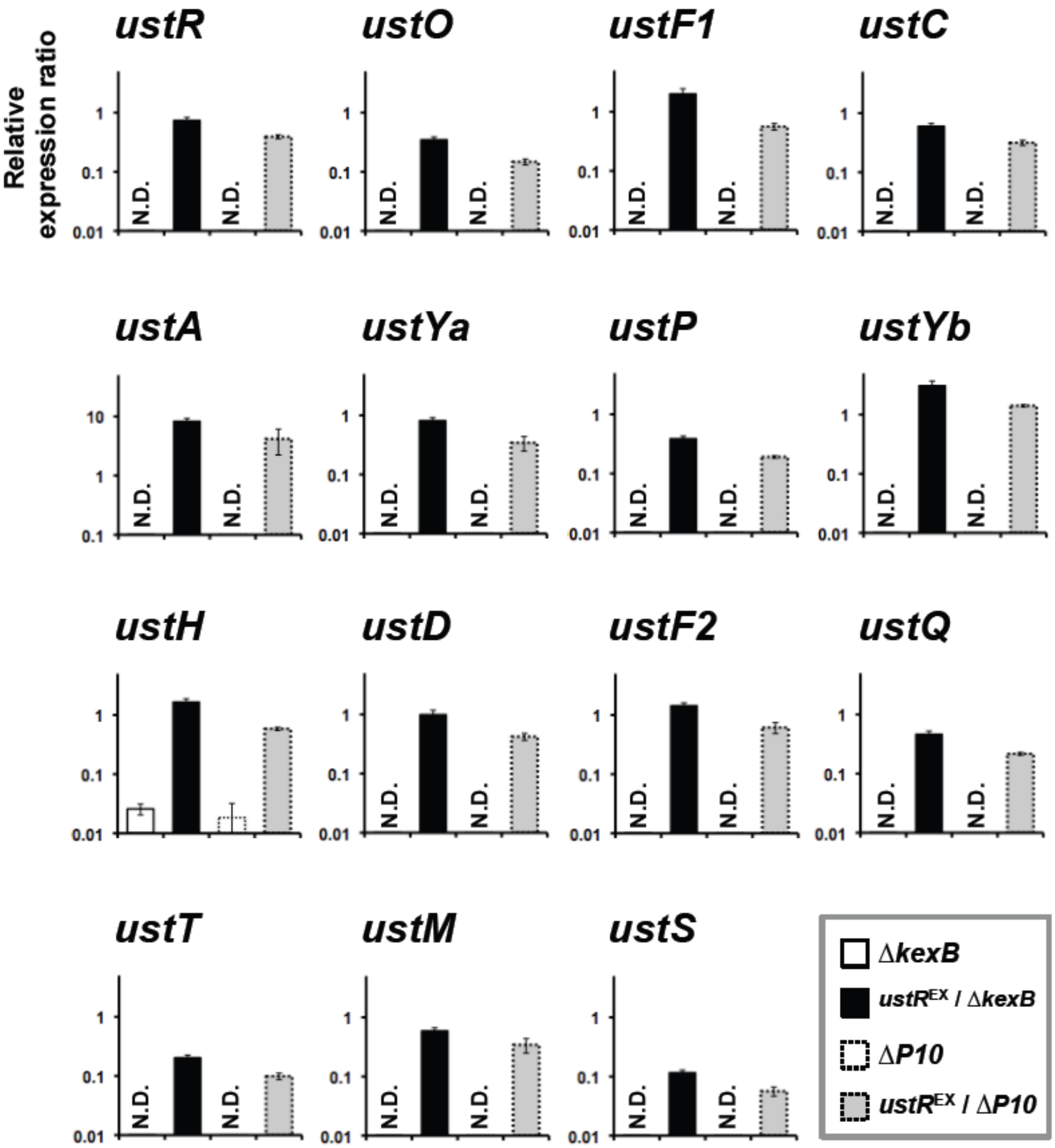

Figure S4 Expression of the genes of the cluster for ustiloxin B biosynthesis in the *A. oryzae* strains Δ*kexB*, *ustR*^{EX}/Δ*kexB*, NSlD-ΔP10 (Δ*P10*), and *ustR*^{EX}/ΔP10. The strains were grown in V8 medium at 30 °C for 5 days. The transcription levels of the indicated genes were determined by quantitative RT-PCR performed on total RNA with gene-specific primers (Table S1). Each value represents the ratio of expression to that of the histone H2B gene in each strain. Error bars represent standard deviations (n=3). N.D., not detectable.
